# Correlation between microRNA-21 and sprouty homolog 2 gene expression in multiple myeloma

**DOI:** 10.3892/mmr.2015.3280

**Published:** 2015-01-29

**Authors:** JIN-HANG WANG, WEN-WEN ZHENG, SHI-TONG CHENG, BO-XIN LIU, FU-RONG LIU, JIAN-QING SONG

**Affiliations:** Laboratory Department of the First Affiliated Hospital of China Medical University, China Medical University School of Medicine Based in Shenyang, Teaching and Research Section of Cell Biology, Shenyang, Liaoning 110001, P.R. China

**Keywords:** microRNA-21, multiple myeloma, sprouty homolog 2, regulation

## Abstract

The aim of the present study was to investigate the expression level of microRNA 21 (miR-21) in the peripheral blood of patients with multiple myeloma (MM) and to investigate the correlation between miR-21 and sprouty homolog 2 (SPRY2) gene expression levels in MM. A total of 30 patients with MM, 15 with monoclonal gammopathy of undetermined significance (MGUS) and 20 normal control (NC) outpatients were selected for the detection of miR-21 and SPRY2 expression using reverse transcription-quantitative polymerase chain reaction. In addition, western blot analysis was performed to detect the expression of miR-21 and SPRY2 in MM cell lines. The expression of miR-21 in U-266 cells following lipofectamine transfection of fluorescence-labeled miR-21 mimic/inhibitor was observed using a fluorescence microscope and the expression level of SPRY2 in the miR-21 mimic/inhibitor-transfected U-266 cells was detected using western blot analysis. The miR-21 expression level in the circulating serum of the MM patient group was significantly higher (P<0.01) than that of the MGUS and NC groups. The MM cell lines with high endogenous miR-21 expression exhibited an expression level of SPRY2 that was significantly lower than that in the MM cells with low endogenous miR-21 expression. The transfection efficiency of fluorescence-labeled miR-21 mimic/inhibitor was >90%. Compared with the miR-21 expression level in untreated U-266 cells (0.82±0.13), the expression level of miR-21 was increased by 120.2-fold in miR-21 mimic-transfected cells (98.6±14.2; P<0.001) and was decreased by 61.9% in the miR-21 inhibitor-transfected cells (0.37±0.06; P<0.05). The grayscale value of protein bands demonstrated that SPRY2 protein expression significantly decreased in miR-21 mimic-transfected U-266 cells compared with that in the inhibitor-transfected, siRNA-transfected and untreated cells (P<0.01). miR-21 may represent a negative regulator involved in the downregulation of SPRY2 in MM. miR-21 is closely associated with the pathogenesis, progression and prognosis of MM and may thus be used as an indicator of poor MM prognosis.

## Introduction

Multiple myeloma (MM) is a type of plasma cell malignancy, which has a relatively high incidence rate among hematological malignancies. MM accounts for ~2% of all cancer-associated fatalities (1) and is exhibiting an increasing trend. Currently, the pathogenesis of MM is hypothesized to involve a progressive process in which monoclonal gammopathy of undetermined significance (MGUS) develops, later progresses to MM in select cases, and eventually evolves into extramedullary myeloma (plasma cell leukemia) in a proportion of patients. Mutations in multiple genes in patient bone marrow plasma cells have been observed throughout this process (2). MicroRNAs (miRNA) are a class of small non-coding RNA molecules ~22 nucleotides in length. As a member of the miRNA family, miRNA-21 (miR-21) is closely associated with tumors and may regulate the expression of sprouty homolog 2 (SPRY2) (3–4). SPRY2 is a specific signaling pathway inhibitor and a member of the Sprouty (SPRY) family. SPRY proteins have four subtypes, SPRY1, 2, 3 and 4. Human SPRY2 consists of 315 amino acid residues (35 kDa), with cysteine-rich residues 178–282 located on the C terminus. Due to its distinct biological effects, SPRY2 has become a focus of intense research. Lao *et al* (5) found that the inhibitory effect of SPRY2 on the receptor tyrosine kinase signaling pathway is significantly increased compared with that of other subtypes. It has been reported (5–8) that miR-21 may regulate the expression of the inhibitory factor SPRY2 of the mitogen-activated protein kinase/extracellular signal-regulated kinase (MAPK/ERK) signaling pathway. In the present study, the expression of miR-21 and SPRY2 in myeloma cells was investigated and the results lay the foundation for the identification of the association of miR-21 and SPRY2 expression with the pathogenesis, progression and malignant characteristics of myeloma, as well as for the clarification of the transcriptional regulatory mechanism of miR-21 in myeloma cells.

## Materials and methods

### Clinical samples

A total of 30 serum samples were obtained from patients with MM, including 16 with κ-type myeloma, 12 with λ-type myeloma and two with non-secretory myeloma. According to the International Staging System (ISS), 13 patients had stage I myeloma, eight had stage II myeloma, and nine had stage III myeloma. The patients in the MM group included 17 males and 13 females, aged between 34 and 86 years, with a mean age of 61.7±12.1 years. There were 15 MGUS serum samples from nine male and six female patients, aged between 35 and 85 years, with a mean age of 60.7±13.2 years. All samples were obtained from outpatients and inpatients in the First Affiliated Hospital of China Medical University (Shenyang, China) between July 2010 and October 2012. MM and MGUS were diagnosed using the 2012 MM diagnostic criteria of the International Myeloma Working Group as a reference (9). There were 20 serum samples from the normal control group (NC group), including 12 male and eight female outpatients without any detectable bone marrow abnormalities that were aged between 35 and 82 years, with a mean age of 59.8±10.6 years. Patients with autoimmune diseases and malignant tumors that may have affected miR-21 expression were excluded from the present study. The study was approved by the ethics committee of the First Affiliated Hospital of China Medical University. Written informed consent was obtained from the patients or their families.

### Cell lines and cell culture

U-266, KM3 and RPMI 8226 human myeloma cell lines were provided by the Cell Biology Laboratory of China Medical University (Shenyang, China). The U-266, KM3 and RPMI 8226 myeloma cell lines were cultured and passaged in RPMI-1640 medium (Hyclone, Thermo Fisher Scientific, Logan, UT, USA) containing 10% fetal bovine serum in a humidified incubator at 37°C with 5% CO_2_. The cells at the logarithmic growth phase were harvested for the subsequent experiments when the cells reached 80% confluence.

### Detection of miR-21 expression

Venous blood was collected from all subjects in morning fasting-state and 5 ml of blood was collected and centrifuged at 950 × g for 10 min prior to use. Reverse transcription-quantitative polymerase chain reaction (RT-qPCR) of miR-21 was performed according to the methods used by Chen *et al* (10). Serum (200 *μ*l) was used to extract total RNA (1 *μ*g) and preserved at −80°C. Total RNA was subsequently subjected to cDNA synthesis using a ReverTra Ace Kit (Promega Corp., Madison, WI, USA). The PCR conditions were as follows: 10 min denaturation at 95°C, followed by 40 two-step cycles of 15 sec at 95°C, 15 sec at 55°C and 20 sec at 72°C; and the reaction mixture was 20*μ*l. The average Ct value of miR-21 was recorded according to the comparative threshold method outlined by Schmittgen and Livak *et al* (11), represented by 2^−ΔΔCt^. ΔΔCt = Experimental group (Ct_target gene_−Ct_housekeeping gene_) − control group(Ct_target gene_−Ct_housekeeping gene_). The experiments were repeated three times. The following primers (Shanghai GenePharma Co.,Ltd) were used: U6 forward, 5′-ATTGGAACGATACAGAGAAGATT-3′ and reverse, 5′-GGAACGCTTCACGAATTTG-3′; miR-21 forward, 5′-ACGTTGTGTAGCTTATCAGACTG-3′ and reverse, 5′-AATGGTTGTTCTCCACACTCTC-3′.

### Detection of protein expression using western blot analysis

Cells were harvested in the logarithmic growth phase at 80% confluence. The cells were lysed in radioimmunoprecipitation analysis lysis buffer (Beyotime Institute of Biotechnology, Shanghai, China) supplemented with protease inhibitors to extract the total cellular protein. The protein concentration was quantified using a bicinchoninic acid protein assay kit (Sigma-Aldrich, St. Louis, MO, USA). The sample was added to 5X loading buffer (Beyotime Institute of Biotechnology), boiled at 100°C for 5 min, and cooled prior to loading. The samples were subject to 10% SDS-polyacrylamide gel electrophoresis, transferred onto nitrocellulose membranes (Takara Bio Inc., Otsu, Japan) and blocked in 5% skimmed milk, whilst agitated for 1 h on a shaker. Following the addition of primary antibodies Sgol (1:100; Shanghai Ximei Chemical Co., Ltd, Shanghai, China) and monoclonal GAPDH (1:5,000; Abbkine, Inc., Redlands, CA, USA), the membranes were sealed within a bag and incubated on the shaker at a speed of 60 rpm at 4°C overnight. The membrane was then washed with phosphate-buffered saline with Tween-20 (PBST) three times for 10 min each time. Following the addition of horseradish peroxidase-labeled goat anti-mouse immunoglobulin M antibody (Luoyang Baitaike Biotechnology Co., Ltd, Luoyong, China; 1:5,000), the membrane was incubated at room temperature for 1 h and then washed with PBST three times, followed by visualization using enhanced chemiluminescence and gel imaging using a gel documentation system (Bio-Rad Laboratories, Inc., Hercules, CA, USA). The quantification of target proteins was accomplished by calculating the relative band intensity in gray-scale images of the proteins (miR-21/GAPDH or SPRY2/GAPDH).

### Transfection of miR-21 mimics/inhibitor

U-266 cells were subject to Lipofectamine™ 2000-mediated G418 transfection (Baiao Biotech Inc., Changchun, China). The cells in the logarithmic growth phase cultured in complete medium were seeded into 12-well plates at a density of 1×10^5^ cells/ml. The cells were divided into four groups: miR-21 mimic group [transfected with miR-21 mimics (Biomics Biotechnologies Co., Ltd, Nantong, China)], miR-21 inhibitor group [transfected with miR-21 inhibitor (Biomics Biotechnologies Co., Ltd)], untreated group (non-transfected cells) and siRNA NC group [transfected with siRNA negative control (Biomics Biotechnologies Co.,Ltd)]. Each group included three wells of cells. The DNA concentration for transfection was 100 nmol/l. The cells were switched to normal medium after 6 h of transfection and Dulbecco’s modified Eagle’s medium/serum containing G418 was added 24 h later to obtain clones after selection for two weeks. Observation under a fluorescence microscope (BX51; Olympus, Tokyo, Japan) confirmed a transfection efficiency exceeding 90%. The clones were cultured continuously to collect cells for RT-qPCR quantification.

### Statistical analysis

Data were analyzed using SPSS 17.0 statistical software (SPSS Inc., Chicago, IL, USA). Measurement data were expressed as the mean ± standard deviation and the difference of means between the two groups were assessed using the small sample t-test. P<0.05 was considered to indicate a statistically significant difference.

## Results

### miR-21 expression levels in the different groups

The relative serum miR-21 level of each group was determined using RT-qPCR. The miR-21 level in the circulating serum of the MM group was significantly increased compared with the MGUS and NC groups (P<0.01). The difference in the expression level between the MGUS group and NC group was not statistically significant (P>0.05). According to ISS staging, the circulating level of miR-21 of stage I MM patients was significantly lower than those of stage II and stage III patients and the differences were statistically significant (P<0.05; Table I).

### RT-qPCR detection of miR-21 and SPRY2 gene expression in MM cell lines

**Figure 1:**
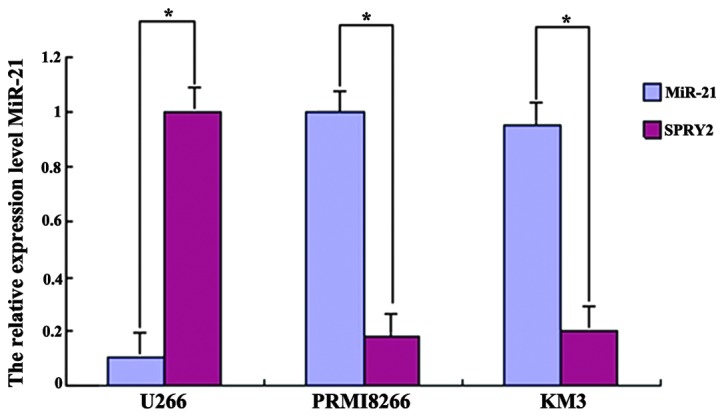
miR-21 and SPRY2 level of gene expression in multiple myeloma cell lines. miR-21, microRNA-21; SPRY2, sprouty homolog 2.

miR-21 and SPRY2 were expressed in the MM cell lines. In the RPMI 8226 and KM3 cell lines, miR-21 was highly expressed and SPRY2 was expressed at a low level. However, in the U-266 cell line, in which a low level of miR-21 was detected, SPRY2 was expressed at a high level. The differences were statistically significant (P<0.01; Fig. 1).

### Western blot analysis detection of miR-21 and SPRY2 expression in MM cell lines

**Figure 2:**
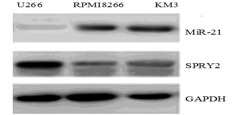
Western blot analysis of miR-21 and SPRY2 gene expression in multiple myeloma cell lines. SPRY2, sprouty homolog 2; miR-21, microRNA-21.

**Figure 3:**
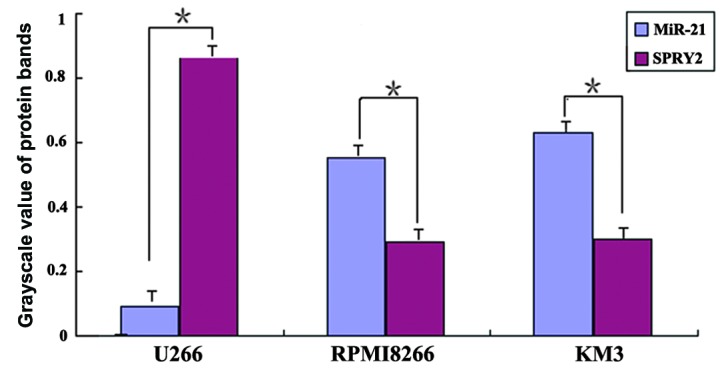
miR-21 and SPRY2 level of protein in multiple myeloma cell lines. U-266 cell line, SPRY2 expression markedly increased; RPMI 8226 and KM3 cell line, SPRY2 expression decreased markedly. ^*^P<0.01. miR-21, microRNA-21; SPRY2, sprouty homolog 2.

Western blot analysis revealed that in the MM cell lines (RPMI 8226 and KM3) with a high level of endogenous miR-21 expression, SPRY2 was expressed at a significantly lower level. By contrast, in the U-266 cell line with low endogenous miR-21 expression, SPRY2 was expressed at a significantly higher level (Fig. 2). The differences in the band intensity of the SPRY2 and miR-21 protein between the different cell lines were statistically significant (P<0.01; Fig. 3).

### Effect of mimics and inhibitor transfection on miR-21 expression in U-266 cells

**Figure 4:**
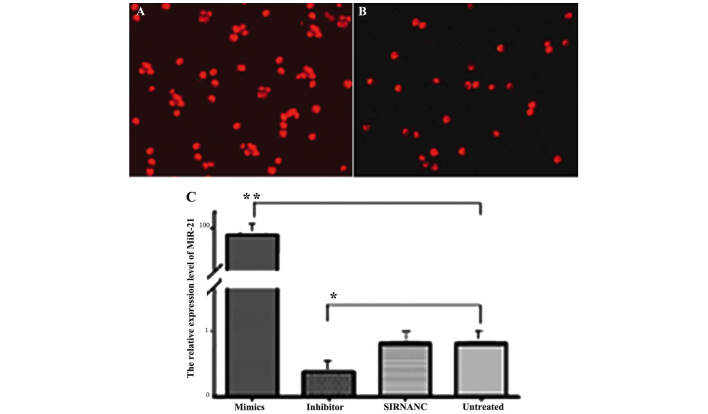
Effects of transfection with mimics and inhibitor on the expression of miR-21 in U-266. (A) miR-21 mimic group and (B) miR-21 inhibitor group immunofluorescence assay, (magnification, ×200). (C) Expression of miR-21 following transfection with mimic/inhibitor. The expression of miR-21 was significantly enhanced following transfection with mimic, ^**^P<0.001; expression of miR-21 decreased following transfection with inhibitor, ^*^P<0.05. miR21, microRNA-21.

U-266 cells were transfected with a fluorescence-labeled miR-21 mimic (Fig. 4A) and inhibitor (Fig. 4B) using liposome and the observation under a fluorescence microscope confirmed a transfection efficiency exceeding 90%. RT-qPCR was used to detect miR-21 expression levels in different groups. The results revealed that the miR-21 level was 98.6±14.2 in the mimic-transfected cells, representing an increase of 120.2-fold compared with that of the non-transfected cells (0.82±0.13); the difference was statistically significant (P<0.001). The expression level was 0.37±0.06 in the inhibitor-transfected cells, a significant decrease of 61.9% compared with the non-transfected cells (P<0.05), whereas no significant difference in miR-21 expression was found between the siRNA NC cells and the non-transfected cells (Fig. 4C).

### Western blot analysis detection of SPRY2 in the miR-21-transfected U-266 cells

**Figure 5:**
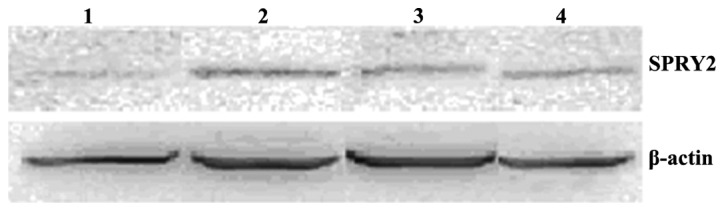
miR-21 regulated the expression of SPRY2 in transfected U266 cells. Lanes: 1, miR-21 mimic; 2, miR-21 inhibitor; 3, siRNA; 4, untreated. miR-21, microRNA-21; siRNA, small interfering RNA; SPRY2, sprouty homolog 2.

After U-266 cells were transfected with miR-21 mimics (50 nM) and inhibitors (100 nM), the band intensity of SPRY2 protein in the miR-21 mimic-transfected cells was significantly lower than that of inhibitor-transfected, siRNA-transfected and untreated cells and the differences were statistically significant (P<0.01; Fig. 5).

## Discussion

MM is an immune system lymphocyte-derived cancer. Due to the annual increases in MM incidence with the aging population, MM has become the second-most common type of hematological malignancy (12–22). The severe humoral immune deficiency and bone damage caused by the excessive production of abnormal plasma cells in the bone marrow and monoclonal immunoglobulin lead to high rates of MM-associated mortality. Currently, the mechanism of MM pathogenesis remains to be elucidated, treatment outcomes exhibit large individual differences and the available prognostic indicators cannot appropriately reflect the complexity of the disease. Therefore, in-depth studies on the mechanism of pathogenesis and prognostic factors may be the key for the development of an effective solution to the clinical problems.

An ideal tumor biomarker should be detected easily and non-invasively. miR-21, an miRNA molecule, which is abnormally expressed in a variety of malignant tissues and has a key role in the regulation of tumorigenesis and progression, is a current area of intense research interest. miR-21 overexpression is closely associated with cancer cell proliferation, metastasis, and disease prognosis of MM, non-Hodgkin’s lymphoma, leukemia and various other non-hematological solid tumors (14–16) miR-21 may regulate the expression of SPRY2. Structurally, the SPRY2 protein (3–5) has a highly conserved, cysteine-rich C-terminus, which may be positioned in the target region of the activated cell membrane, while its N-terminus is highly mutable. Several studies (5,17–19) have demonstrated that the expression of SPRY2 was downregulated or inhibited in prostate cancer, breast cancer and malignant glioma, leading to the deregulation and overactivation of MAPK/ERK signaling in tumor cells. Therefore, SPRY2 is considered to be an oncogene, which inhibits MAPK/ERK signaling. The findings of the present study provided a basis for the further investigation of SPRY2 function, its molecular mechanisms in MM and its clinical significance.

The serum miR-21 levels in each group were determined using RT-qPCR and it was observed that miR-21 was over-expressed in patients with MM. Serum circulating miR-21 levels in the MM group were significantly higher than those in the MGUS group and NC group (P<0.01), indicating a close correlation of miR-21 with the pathogenesis and progression of MM, which is consistent with findings previously reported in the literature (20–22). A study (23) reported that the expression of miR-21 is significantly higher in myeloma cells than in the plasma cells of MGUS and NC groups, suggesting a positive correlation between circulating miR-21 levels and the tumor cell load in patients. As the high level of serum circulating miR-21 in MM patients is likely to be due to myeloma cells, the serum circulating miR-21 level may be used to monitor MM progression and to evaluate therapeutic performance. The level of circulating miR-21 in the MGUS group was higher than in the NC group; however, the difference was not statistically significant (P>0.05); a larger sample size is required for further investigation.

The present results revealed low or absent expression of SPRY2 in the RPMI 8266 and KM3 MM cell lines, which may be associated with the inhibition of SPRY2 expression by the high level of miR-21 in MM cells. Using SPRY2 over-expression (24), certain studies have reported that SPRY2 partially inhibits the activation of the MAPK/ERK signaling pathway and inhibits interleukin 6 (IL-6)-induced MM cell proliferation, suggesting that SPRY2 has a role in tumor suppression in MM cells through the inhibition of the activation of MAPK/ERK signaling (5,17–19). The downregulation of miR-21 by inhibitor transfection may increase the expression of SPRY2 in the U-266 MM cell line, suggesting that miR-21 may regulate SPRY2 expression.

Furthermore, using western blotting, it was identified that the expression level of SPRY2 was significantly lower in the RPMI 8226 and KM3 MM cell lines, which have high endogenous miR-21 expression levels, whereas the level of SPRY2 was significantly higher in the U-266 cell line with low endogenous miR-21 expression. The negative correlation between miR-21 and SPRY2 was further confirmed by the observation that the expression of SPRY2 significantly decreased in the stably-transfected MM cell line overexpressing exogenous miR-21.

In conclusion, the present study preliminarily confirmed that miR-21 is likely a negative regulatory factor causing downregulation of SPRY2 in MM. miR-21 is closely associated with the pathogenesis, progression and prognosis of MM, and thus may be used as an indicator of poor prognosis in MM patients. With the development and application of miRNA antisense oligonucleotide technology, miR-21 may become a biomarker for MM diagnosis and a novel target for the treatment of drug resistance.

## Figures and Tables

**Table I:** Circulating miR-21 in all groups

Variables	n	miR-21
Group		
MM	30	2.38±0.32^b^
MGUS	15	0.77±0.20
Normal control	20	0.43±0.13
ISS installments		
I	13	0.86±0.19^a^
II	9	2.79±0.64
III	12	2.88±0.52

a P<0.05, compared with II and III;

b P<0.01, compared with MGUS and normal controls. miR-21, microRNA-21; MM, multiple myeloma; MGUS, monoclonal gammopathy of undetermined significance; ISS, International Staging System.
